# Evolution of dental tissue mineralization: an analysis of the jawed vertebrate *SPARC* and *SPARC-L* families

**DOI:** 10.1186/s12862-018-1241-y

**Published:** 2018-08-30

**Authors:** Sébastien Enault, David Muñoz, Paul Simion, Stéphanie Ventéo, Jean-Yves Sire, Sylvain Marcellini, Mélanie Debiais-Thibaud

**Affiliations:** 1 Institut des Sciences de l’Evolution de Montpellier, ISEM, Univ Montpellier, CNRS, IRD, EPHE, Université Montpellier, UMR5554 Montpellier, France; 2 Laboratory of Development and Evolution, Department of Cell Biology, Faculty of Biological Sciences, University of Concepción, Concepción, Chile; 3 Institute for Neurosciences of Montpellier, Institut National de la Santé et de la Recherche Médicale, U1051 Montpellier, France; 4 Institut de Biologie Paris-Seine, Université Pierre et Marie Curie, UMR7138 Evolution Paris-Seine, Paris, France

**Keywords:** Odontodes, Gnathostomes, SPARC/SPARC-L l, Fibrillar collagens, Enamel, Enameloid

## Abstract

**Background:**

The molecular bases explaining the diversity of dental tissue mineralization across gnathostomes are still poorly understood. Odontodes, such as teeth and body denticles, are serial structures that develop through deployment of a gene regulatory network shared between all gnathostomes. Dentin, the inner odontode mineralized tissue, is produced by odontoblasts and appears well-conserved through evolution. In contrast, the odontode hypermineralized external layer (enamel or enameloid) produced by ameloblasts of epithelial origin, shows extensive structural variations. As *EMP* (Enamel Matrix Protein) genes are as yet only found in osteichthyans where they play a major role in the mineralization of teeth and others skeletal organs, our understanding of the molecular mechanisms leading to the mineralized odontode matrices in chondrichthyans remains virtually unknown.

**Results:**

We undertook a phylogenetic analysis of the SPARC/SPARC-L gene family, from which the EMPs are supposed to have arisen, and examined the expression patterns of its members and of major fibrillar collagens in the spotted catshark *Scyliorhinus canicula*, the thornback ray *Raja clavata*, and the clawed frog *Xenopus tropicalis*. Our phylogenetic analyses reveal that the single chondrichthyan *SPARC-L* gene is co-orthologous to the osteichthyan *SPARC-L1* and *SPARC-L2* paralogues. In all three species, odontoblasts co-express *SPARC* and collagens. In contrast, ameloblasts do not strongly express collagen genes but exhibit strikingly similar *SPARC-L* and *EMP* expression patterns at their maturation stage, in the examined chondrichthyan and osteichthyan species, respectively.

**Conclusions:**

A well-conserved odontoblastic *collagen/SPARC* module across gnathostomes further confirms dentin homology. Members of the *SPARC-L* clade evolved faster than their *SPARC* paralogues, both in terms of protein sequence and gene duplication. We uncover an osteichthyan-specific duplication that produced *SPARC-L1* (subsequently lost in pipidae frogs) and *SPARC-L2* (independently lost in teleosts and tetrapods).Our results suggest the ameloblastic expression of the single chondrichthyan *SPARC-L* gene at the maturation stage reflects the ancestral gnathostome situation, and provide new evidence in favor of the homology of enamel and enameloids in all gnathostomes.

**Electronic supplementary material:**

The online version of this article (10.1186/s12862-018-1241-y) contains supplementary material, which is available to authorized users.

## Background

Dentin and enamel are found in serially developing skeletal organs called odontodes which include oral and pharyngeal teeth, dermal scales and denticles [1–3]. Stem-gnathostome fossils suggest that initially odontodes covered the body and were subsequently recruited to the mouth region [1, 2, 4, 5]. The early morphogenesis of all odontodes is initiated by similar epithelial-mesenchymal interactions and relies on a well-conserved genetic cascade in extant gnathostomes [6–14]. Dentine, the inner odontode matrix, is evolutionarily conserved in terms of tissue structure, while structural variation has been described for the odontode outer mineralized region, mostly known as enamel or enameloid. Enamel was originally described as a sarcopterygian-specific tissue exclusively produced by ameloblasts, devoid of collagen fibers, and clearly demarcated from the underlying dentin produced by odontoblasts [6, 15]. By contrast, enameloid is produced by odontoblasts (with or without an ameloblastic contribution), is continuous with dentin, and is characterized by variable degrees of collagen contents [6, 15, 16]. Furthermore, in caudate amphibian and teleost species harboring enameloid-covered odontodes, ameloblasts express type I fibrillar collagens [17–20].

A body of literature has led to two incompatible views of odontode evolution. On the one hand, it has been proposed that the osteichthyan last common ancestor harbored enameloid-covered odontodes [2, 21]. This is because (i) enameloid is present at the surface of stem gnathostome odontodes, chondrichthyan teeth and dermal denticles, actinopterygian oral and pharyngeal teeth, and caudate amphibian larval teeth [22–25] and (ii) teleost and chondrichthyan enameloids were proposed to be homologous [2, 21]. On the other hand, it has been inferred that the osteichthyan last common ancestor harbored enamel-covered odontodes [2, 26–29]. Indeed, evidence showing that (i) *EMP* (Enamel Matrix Proteins) genes are specifically expressed in ameloblasts during mammalian enamel matrix secretion and maturation, [30] and (ii) *EMPs* are present in the gar genome [4], suggest that the surface tissue of tooth and ganoid scales of non-teleost actinopterygians (polypterids and lepisosteids, [26, 27]) are homologous to the sarcopterygian enamel [4]. These data led to the prediction that type I fibrillary collagens will be expressed in chondrichthyan ameloblasts [2], together with EMP-related genes belonging to the SCPP (secretory calcium-binding phosphoproteins) gene family.

The SPARC (secreted protein acidic and rich in cysteine), SPARC-L1 (SPARC-Like1) and SCPP proteins are crucial extracellular matrix components involved in several major vertebrate innovations [17, 20, 31–33]. The classical evolutionary scenario posits that the *SPARC-L1* and *SPARC* paralogues arose from two rounds of vertebrate-specific genome duplications [34, 35]. The gar and coelacanth *SPARC-L1* genomic loci were recently shown to contain a *SPARC-L2* gene of unknown evolutionary history [5, 36]. The *SCPP* gene family has as yet only been found in osteichthyan vertebrates, and is thought to have originated through a series of tandem duplications of the *SPARC-Like1* (*SPARC-L1*) gene [5, 32, 37–40]. The *SCPP* family includes P/Q-rich protein genes (such as the EMPs) and acidic, bone and dentin protein genes also known as small integrin-binding ligand, N-linked glycoproteins (SIBLING) genes [5, 20, 34, 41, 42]. SPARC-L1 and SPARC are extracellular proteins harboring collagen-interacting and calcium-binding domains, thereby contributing to matrix deposition and mineralization [35, 43–45]. Accordingly, both *SPARC* and type I fibrillar collagen genes are highly expressed in osteichthyan odontoblasts [20, 43, 46–48]. All examined osteichthyan *EMP* and *SIBLING* members are expressed by odontoblasts and/or ameloblasts [17, 20, 49, 50], and code for extracellular regulators of dentin (e.g. *Dmp-1*, a SIBLING member) or enamel (e.g. *Amtn* and *Enam*, two EMP members) mineralization [51–53]. A high rate of gene gain, loss and divergence has dramatically modified the *SCPP* repertoire in distinct osteichthyan lineages, blurring orthology relationships [5, 17, 20, 32–34, 36, 54]. Nevertheless, a few conserved *EMP* genes can unambiguously be identified in sarcopterygians and actinopterygians, as is the case of *Enam* and *Ambn* [5, 36, 41]. Changes in the *SCPP* gene content led to proposals that this causes odontode matrix structural variations in osteichthyans [17, 20, 34]. However, no *SCPP* gene has been detected in the elephant shark genome so far, which only seems to harbor putative *SPARC* and *SPARC-L1* orthologues [55].

In this study, we sought to improve understanding of jawed vertebrate odontode evolution by (i) clarifying the evolutionary relationships between *SPARC*, *SPARC-L1* and *SPARC-L2*, (ii) determining whether or not members of the SPARC and SPARC-L gene families are specifically expressed by ameloblasts and/or by odontoblasts in chondrichthyans, using embryos from the spotted catshark *Scyliorhinus canicula* (*S.c.*) and the thornback ray *Raja clavata* (*R.c.*), (iii) evaluating whether type I fibrillar collagens are expressed in ameloblasts in *S.c.*, *R.c.* and in the osteichthyan *Xenopus tropicalis* (*X.t.*), and (iv) examining SPARC gene expression in odontodes of a species lacking both *SPARC-L1* and *SPARC-L2*, an extremely rare situation which has so far only been reported in the *Xenopus* genus.

## Results

### Reconstruction of SPARC/SPARC-L1/−L2 gene phylogeny

**Fig. 1:**
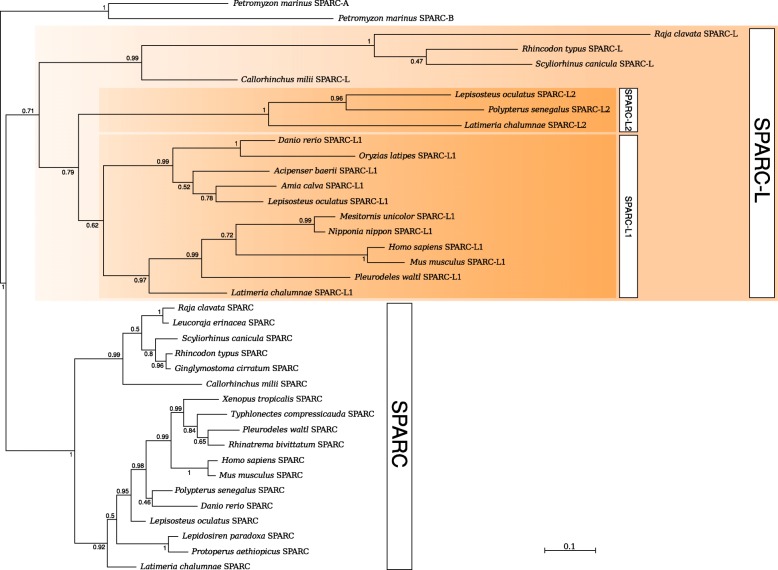
Phylogenetic relationships of the vertebrate SPARC and SPARC-L gene families. Bayesian inference was run with Phylobayes and the site-heterogeneous CAT+Γ4 model of sequence evolution. The tree was rooted with the lamprey SPARC-A and SPARC-B sequences. Posterior probabilities are indicated on the branches

We performed phylogenetic analyses of the SPARC and SPARC-L1/−L2 homologous protein sequences obtained in public databases, as well as the 13 novel sequences identified in jaw transcriptomes [56], including two isolated transcripts from the lesser spotted catshark and the thornback ray (Additional file 1). As outgroups sequences, we used the orthologous protein from two amphioxus species, and the lamprey SPARC-A and SPARC-B sequences (Additional file 1). Our alignment allowed phylogenetic reconstructions based on 235 amino-acid positions (Fig. 1 and Additional file 2: a-c). Branch supports were higher when the amphioxus sequences were excluded from the analyses, both for Bayesian (compare Fig. 1 and Additional file 2: a) and Maximum Likelihood reconstructions (compare Additional file 2: b and c). All phylogenetic reconstructions led to a maximal support of a SPARC clade (Fig. 1 and Additional file 2: a-c) including both the chondrichthyan and the osteichthyan SPARC sequences. A lesser resolution within the osteichthyan SPARC sequences was probably due to a lack of phylogenetic signal at this level of the tree.

The evolutionary history of the gnathostome SPARC-L1/−L2 clade was more difficult to decipher from these analyses (Fig. 1). Indeed, in addition to SPARC-L1, both the spotted gar and coelacanth harbored a second paralogue which had previously been named “SPARCL2”, “SPARCL1-like”, “SPARCL1L1” or “SPARCL1A” [5, 32, 35, 36]. In agreement with Qu et al. [5] and based on the phylogenetic arguments described below, we referred to these gar and coelacanth long-branch copies as “SPARC-L2”, in opposition to the osteichthyan SPARC-L1 sequences which were found in more species and comparatively evolved at a slower rate (Fig. 1). Here we identified a new SPARC-L2 sequence from the bichir (*Polypterus senegalus*) jaw transcriptomic data (Fig. 1). The monophyly of the osteichthyan SPARC-L2 group was robust in all analyses (Fig. 1 and Additional file 2: a-c). This SPARC-L2 clade consistently clustered with the osteichthyan SPARC-L1 group in Bayesian reconstructions, albeit with medium robustness (posterior probability 0,79, see Fig. 1, also Additional file 2: a). Both ML trees were congruent with this clade, but with weak support (Additional file 2: b-c). In all analyses, the catshark, thornback ray, whale shark and elephant shark homologues clustered together as the sister group to the osteichthyan SPARC-L1/SPARC-L2 clade, although with medium to weak support (Fig. 1 and Additional file 2: a-c). This topology strongly supports the idea that an osteichthyan-specific gene duplication led to the SPARC-L1 and SPARC-L2 copies, which directly implies that the use of the *SPARC-L1* name should be avoided for the chondrichthyan genes. We therefore respectively named *Sc-SPARC-L* and *Rc-SPARC-L* the spotted catshark and thornback ray genes related to the osteichthyan clade that groups the *SPARC-L1* and *SPARC-L2* genes together.

### Expression patterns of *SPARC* and *SPARC-L* genes in developing teeth and dermal denticles

**Fig. 2:**
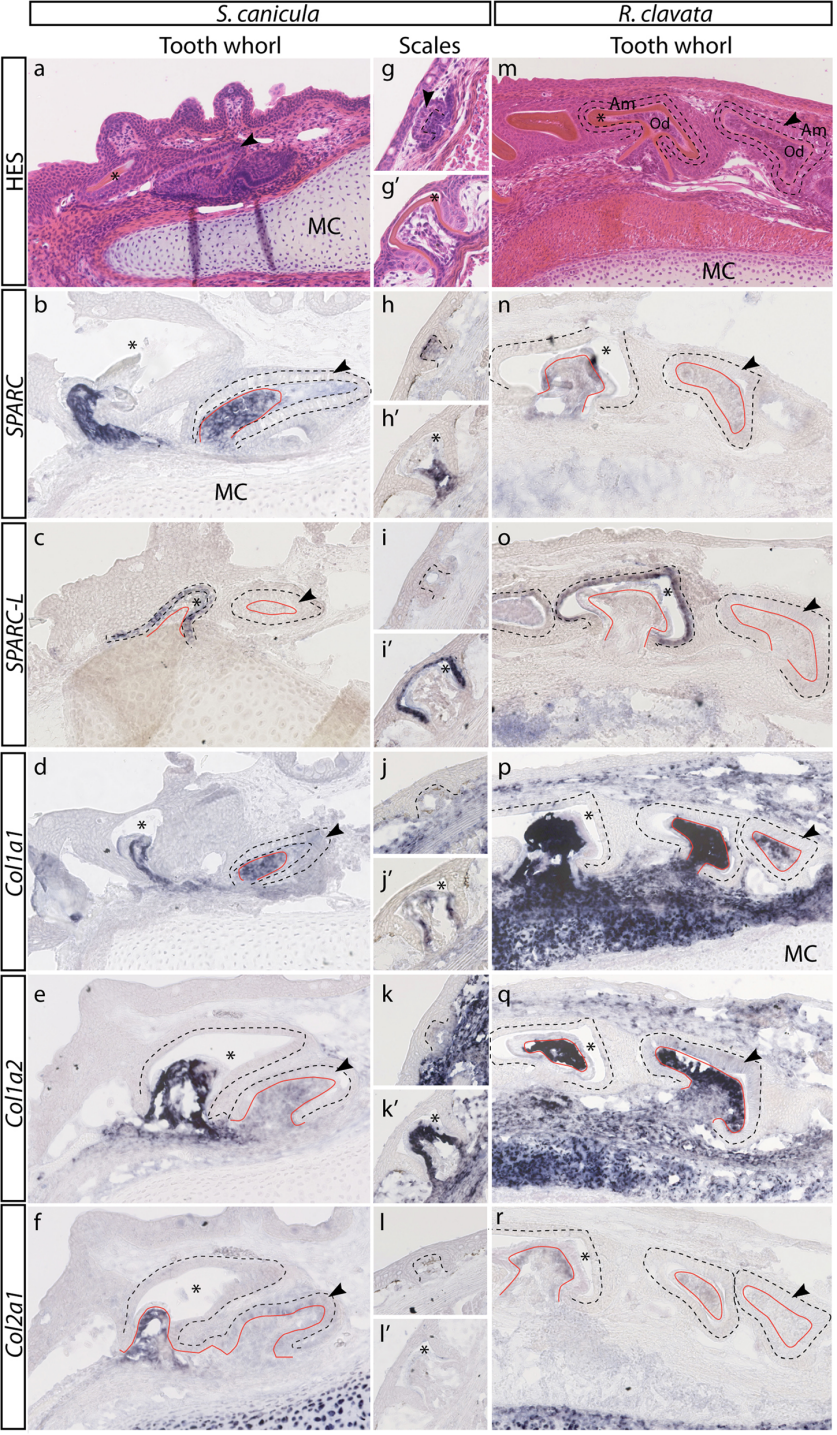
Histology and gene expression in the developing odontodes of *Scyliorhinus canicula* and *Raja clavata*. Lower jaw longitudinal sections (anterior, left; dorsal, up) of a 20 cm long *S.c.* juvenile (**a**), a 9 cm long *S.c.* embryo (**b**-**f**), and a 9 cm long *R.c* embryos (**m**-**r**), revealing the presence of tooth series at the maturation stage as well as less developed secretory stage tooth bud harboring columnar ameloblasts (black arrowhead). Thoracic transverse sections are shown for of 6 cm (**g**-**l**) and 7 cm (**g**’-**l’**) long *S.c.* embryos, focusing on developing primary dorsal dermal denticles at late morphogenesis and late maturation stage, respectively (dorsal to the top). Sections were stained with HES (**a**, **g**, **g’**, **m**) or in situ hybridized against *SPARC* (**b**, **h**, **h’**, **n**), *SPARC-L* (**c**, **i**, **i’**, **o**), *Col1a1* (**d**, **j**, **j’**, **p**), *Col1a2* (**e**, **k**, **k’**, **q**) and *Col2a1-L* (**f**, **l**, **l’**, **r**). The asterisks locate the mineralized matrix in teeth and denticles at the late mineralization stage, separating the ameloblasts (Am, located by the dashed lines) from the odontoblasts (Od, delineated by the orange line). MC: Meckel. The scale bar in (**a**) represents 100 μm in (**a**-**f**, **m**-**r**), and the scale bar in (**g**) represents 50 μm in (**g**-**l’**)

**Fig. 3:**
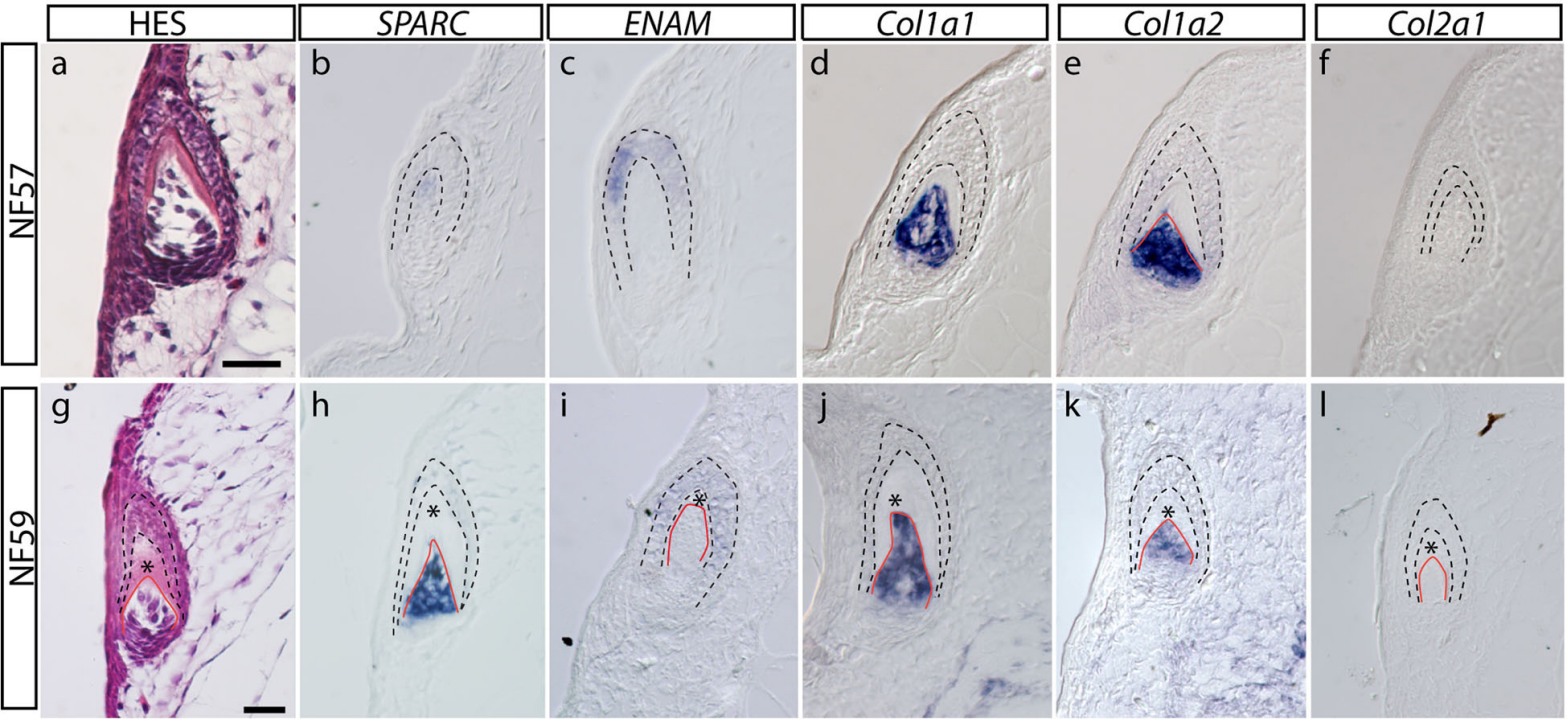
Histology and gene expression in developing teeth of *Xenopus tropicalis*. Longitudinal sections of the *X.t.* upper jaw at the NF57 developmental stages were stained with HES (**a**, **g**) or processed by in situ hybridization for the indicated genes (b-f and h-l). Ameloblasts and odontoblasts are delineated by black dotted lines or by an orange line, respectively. The asterisks locate the mineralized matrix. The scale bars in (**a**) and (**g**) respectively represent 20 μm in (**a**-**f**) and (**g**-**l**)

*SPARC* and *SPARC-L* gene expression was studied in the lesser spotted catshark (*S.c.*) and thornback ray (*R.c.*) odontodes (Fig. 2). *SPARC* and *Enam* (an EMP gene) expression was studied in the frog (*X.t.*, Fig. 3).

In the catshark, the early stage of dentin and enameloid matrix synthesis could be identified by the presence of columnar ameloblasts at the tooth and denticle surface (secretory stage: Fig. 2a and g), while later tooth and denticle buds were covered with cubic ameloblasts facing significant amount of mineralized matrix (maturation stage: Fig. 2a and g’). At all developmental stages, the Sc-*SPARC* expression was restricted to odontoblasts in both teeth and dermal denticles (Fig. 2b, h, h’). In contrast, Sc-*SPARC-L* was expressed only in the ameloblasts in both developing teeth and dermal denticles at the maturation stages (Fig. 2c and i’). At the earlier secretory stage, ameloblasts did not display any detectable expression of the *SPARC-L* transcripts (Fig. 2c and i).

In the thornback ray, most dermal denticles develop as massive dermal thorns, making it technically challenging to obtain denticle sections at different stages (but see ref. [57]). We therefore focused on tooth development only. Longitudinal sections of the thornback ray lower jaw showed developing teeth at the secretory and maturation stages, similar to the catshark observations (compare Fig. 2a and m). We detected a faint Rc-*SPARC* expression in the tooth bud mesenchyme (Fig. 2n). In contrast, Rc-*SPARC-L* was clearly expressed in the inner epithelial cell-layer (ameloblasts), exclusively at the maturation stage when secretion was well advanced and ameloblasts have lost their secretory morphology (Fig. 2o).

To date, only the *Xenopus* genus (*X. laevis* and *X. tropicalis*) has been reported to have lost both the *SPARC-L1* and *SPARC-L2* paralogues, a unique situation amongst osteichthyans [20]. In *Xenopus tropicalis*, *SPARC* expression has previously been described during embryogenesis and osteogenesis, but not during odontogenesis [35, 58]. To evaluate whether the *SPARC-L1* and *SPARC-L2* loss was accompanied by compensatory mechanisms that might have involved changes in the expression domain of other related genes, we examined *SPARC* expression in developing teeth of *Xenopus tropicalis.* We find that, at stages NF57 and NF59, Xt-*SPARC* expression was restricted to the odontoblasts (Fig. 3a, b, g, h). For the sake of comparison with chondrichthyan *SPARC-L* expression, we chose to also examine the expression pattern of *Enam*, an EMP gene, whose expression had not been reported in *Xenopus* so far. We showed that Xt-*Enam* is expressed specifically in the ameloblasts at both developmental stages (Fig. 3c, i).

### Expression patterns of type I and II collagen genes in developing teeth and dermal denticles

A phylogeny of the type I and II fibrillar collagen families including chondrichthyan sequences was previously published, leading to the unambiguous identification of the *Col1a1*, *Col1a2* and *Col2a1* paralogues used in the present study [59]. In the catshark, *Sc-Col1a1*, *Sc-Col1a2* transcripts were robustly expressed in secretory odontoblasts, both in developing teeth (Fig. 2d-f) and in dermal denticles at the late morphogenesis stage (Fig. 2j-l’). Sc-*Col2a1* expression was weak in mineralizing tooth buds and undetectable in denticle buds (Fig. 2f, l, l’). *Sc-Col2a1* was also faintly expressed in early tooth buds ameloblasts (Fig. 2f, arrowhead).

In the thornback ray, Rc-*Col1a1* and Rc-*Col1a2* were strongly expressed in putative odontoblasts (Fig. 2p-q). Rc-*Col2a1* was expressed at much lower levels in secretory ameloblasts at early developmental stage of tooth bud development (Fig. 2q, arrowhead).

The absence of expression of type I fibrillar collagen genes in the ameloblasts of these two chondrichthyans stands in sharp contrast with the situation observed in other species with enameloid-covered teeth, such as teleosts, and salamander larvae [17, 18, 20]. Mammalian ameloblasts synthesize an enamel matrix (not enameloid) and are known to be devoid of fibrillar collagen expression. In order to polarize evolutionary change, we examined *Xenopus tropicalis* also with enamel-producing ameloblasts, and found that both *Xt-Col1a1* and *Xt-Col1a2* transcripts were strongly expressed by odontoblasts, yet clearly excluded from ameloblasts, at two distinct stages of tooth development (Fig. 3d, e, j, h). No expression could be detected in developing tooth buds for *Xt-Col2a1* (Fig. 3f, l), while a signal was observed in the head cartilage present on the same section and used as an internal positive control (not shown).

## Discussion

### Evolution of the SPARC-L family

**Fig. 4:**
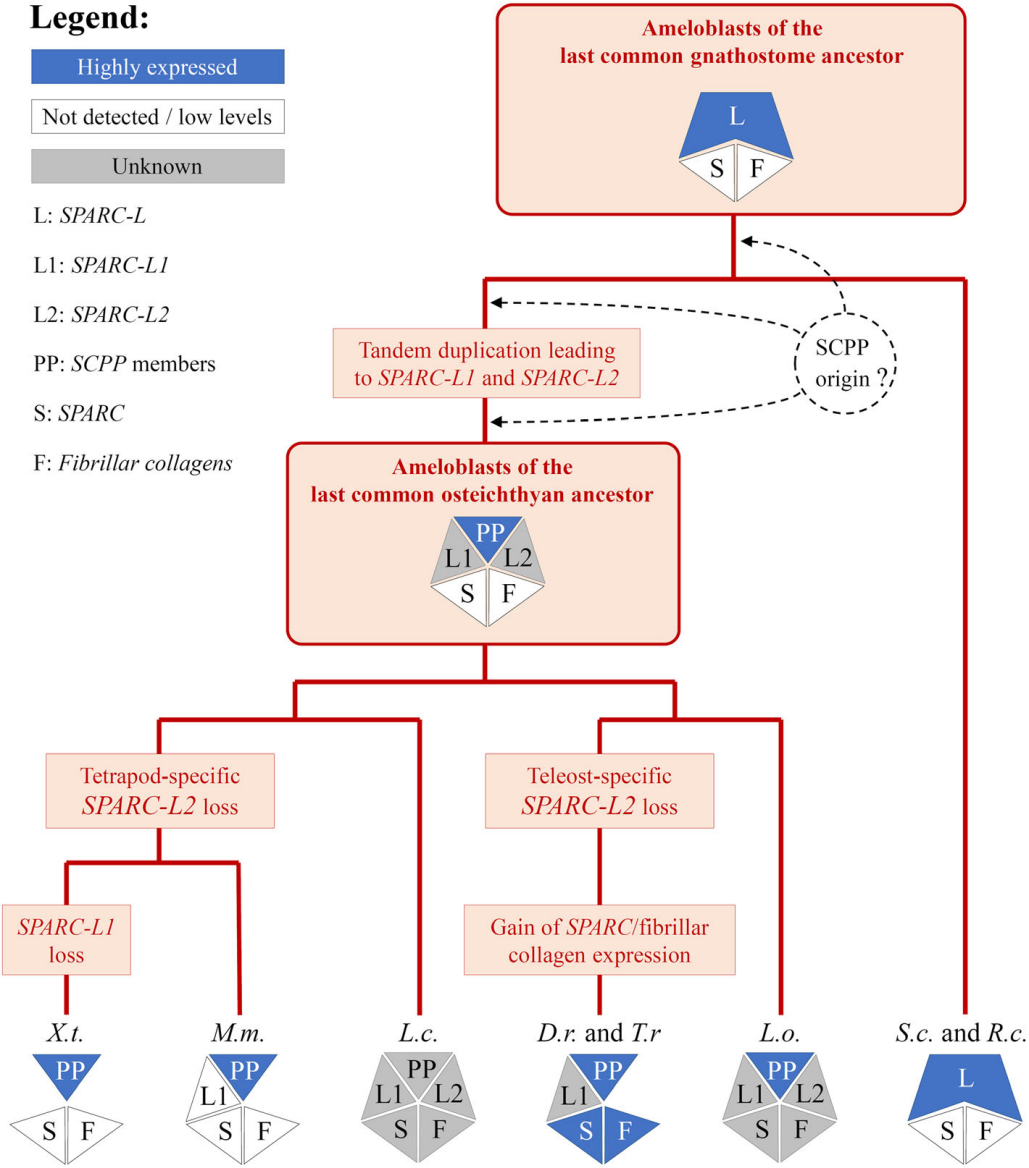
Favored model for the evolution of gene content and ameloblastic expression in gnathostomes. A cladogram shows the classically accepted phylogenetic relationships of *Scyliorhinus canicula* (*S.c.*), *Raja clavata* (*R.c.*), *Lepisosteus oculatus* (*L.o*.), *Danio rerio* (*D.r.*), *Takifugu rubripes* (*T.r.*), *Latimeria chalumnae* (*L.c.*), *Xenopus tropicalis* (*X.t.*) and *Mus musculus* (*M.m.*), as well as putative ancestral situations and polarized evolutionary changes. Pentagons summarize the gene content identified in each species and inferred to have existed in their last common ancestor. Ameloblastic gene expression status is summarized as transcribed (blue), undetected (white) or unknown (grey). Dotted arrows represent unresolved ambiguities with respect to the origin of the SCPP members. See text for details and references

Our phylogenetic analyses suggest a new evolutionary scenario for the expansion of the SPARC-L gene family in gnathostomes. Recent genomic data have shown that *SPARC-L1* and *SPARC-L2* are located close to each other in the gar genome, and that the synteny around both genes is conserved between gar and coelacanth [5, 32, 36]. Here we identified *SPARC-L2* in the bichir jaw transcriptome, but this gene is absent in either tetrapod or teleost genomes. Therefore, *SPARC-L2* has probably been independently lost early in the teleost and tetrapod lineages. We could not identify any SPARC-L2 sequence in the transcriptome of *Lepidosiren paradoxa* or *Protopterus aethiopicus*, suggesting that this copy was lost in the last common ancestor of Dipnoi and Tetrapods. Except in pipidae frogs, *SPARC-L1* is identified in all available osteichthyan genomes. This suggests osteichthyans ancestrally exhibited a repertoire of at least three *SPARC/SPARC-L* paralogues: *SPARC* which is present in all species examined to date, and *SPARC-L1* and *SPARC-L2* which were independently lost in distinct lineages (illustrated in Fig. 4). In chondrichthyans, we identified only two genes in the available transcriptomics and genomics data: the *SPARC* orthologue and one paralogue which we refer here as *SPARC-L* (formerly coined SPARC-L1 [60]). In the elephant shark genome, *SPARC-L* is located in a region of conserved synteny compared to the osteichthyan *SPARC-L1/SPARC-L2* locus [5, 36, 61]. Our phylogenetic analyses support the notion that the chondrichthyan SPARC-L clade is the sister-group to the osteichthyan SPARC-L1/SPARC-L2 clade (Fig. 1), implying that *SPARC-L1* and *SPARC-L2* arose through an osteichthyan-specific tandem duplication of an ancestral gnathostome *SPARC-L* gene. The chondrichthyan *SPARC-L* is therefore co-orthologous to the osteichthyan *SPARC-L1* and *SPARC-L2* genes. As a consequence, the chondrichthyan gene is referred to as “*SPARC-L”* forthwith, as this nomenclature best reflects the evolutionary history of the family (Figs. 1 and 4). In this scenario, the *SPARC* and *SPARC-L* paralogues arose during the two rounds of vertebrate-specific whole genome duplications.

Longer branches reveal that the SPARC-L/SPARC-L1/SPARC-L2 proteins evolve faster than the SPARC sequences, potentially interfering with phylogeny reconstruction and leading to weaker node support (Fig. 1 and Additional file 2). Hence, an alternative hypothesis to the aforementioned scenario is that *SPARC-L* is a highly derived *SPARC-L1* or *SPARC-L2* copy, which duplication was followed by the loss of the other duplicate in all chondrichthyan lineages. Nevertheless, the tree topology, the synteny data, and the presence of a single *SPARC-L* gene in all chondrichthyan transcriptomes and genomes examined to date provide sound arguments in favor of *SPARC-L1* and *SPARC-L2* being of an osteichthyan origin. Interestingly, an accelerated rate of evolution is a shared feature of the *SPARC-L/SPARC-L1/SPARC-L2* and *SCPP* genes [17, 34, 42]. We therefore propose the term “*SPARC-L* gene family” is used to refer to the full repertoire of gnathostome *SPARC-L*, *SPARC-L1*, *SPARC-L2* and *SCPPs*, since these are all derived from a single ancestral gnathostome *SPARC-L* gene. We can conclude that duplicates in the *SPARC-L* family: (1) are found at a single locus of conserved synteny between all examined gnathostome genomes, (2) encode rapidly diverging protein sequences (when compared to their SPARC orthologues), and (3) have been prone to frequent independent gene gains and losses in osteichthyans.

### A conserved collagen/SPARC module

We show that odontoblasts co-express *SPARC*, *Col1a1*, *Col1a2* and *Col2a1* in catshark teeth and dermal denticles, and in thornback ray teeth, thereby supporting the consistency of this co-expression in selachians and the serial nature of chondrichthyan odontodes [9–11]. *SPARC*, *Col1a1* and *Col1a2* are also co-expressed in *X.t.* odontoblasts (this study), as previously reported in teleost and mammalian representatives [17, 20, 47]. These observations confirm the classical hypothesis that the dentin is homologous across jawed vertebrates, and we therefore propose that *SPARC* and type I collagen genes are final targets of the odontoblastic regulatory network active in teeth and dermal denticles [9, 14].

In contrast, while the absence of any detectable ameloblastic expression of the collagen/SPARC module in *S.c.*, *R.c.* and *X.t.* is strikingly similar to the situation reported in mammals [47], it is markedly different from the fugu, zebrafish and salamander expression patterns [17, 18, 20]. Based on this, we propose that, in the gnathostome last common ancestor, the ameloblasts did not express the collagen/SPARC module, and that this character (absence of expression) was subsequently modified in distinct lineages. One possible scenario is that the collagen/SPARC ameloblastic expression originated in the osteichthyan last common ancestor, and that at least two secondary losses occurred, in mammals and in the clawed frog lineage after it separated from salamanders (three evolutionary events). However, in this study, we favor a more parsimonious scenario (Fig. 4) according to which the collagen/SPARC module was recruited twice in odontoblasts, in teleosts and salamander [17, 18, 20].

### *SPARC-L* expression supports the homology of the odontode outer layer

In mice, *SPARC-L1* is broadly expressed and dispensable for development [62–64], and to the best of our knowledge, no detailed *SPARC-L1* and/or *SPARC-L2* expression patterns have been reported in the odontodes of any osteichthyan species. Therefore, the ameloblast-specific expression of the *SPARC-L* gene in elasmobranch teeth and dermal denticles is remarkable and strikingly similar to some of the osteichthyan *SCPP* members. Indeed, we find that in *X.t.*, in which both *SPARC-L1* and *SPARC-L2* have been lost, the EMP gene *Enam* is exclusively expressed in ameloblasts, which is comparable to the previously reported expression of *Amel* and *Odam* in *Xenopus* [20] and of *Amel* and *Amtn* in salamander [18, 65]. There are at least two hypotheses that might account for these ameloblast-specific expression patterns: (i) that they follow two convergent acquisitions in chondrichthyans (for *SPARC-L*) and osteichthyans (for *SCPP*s), or (ii) that the chondrichthyan *SPARC-L* and the osteichyan *SCPP* genes have inherited their ameloblatic-specific expression from the ancestral *SPARC-L* gene. The latter hypothesis (shown in Fig. 4) is well supported if we assume that the osteichthyan *SCPP* genes (particularly the *EMP* members) have largely taken over the function ancestrally fulfilled by SPARC-L. Hence, the SCPP emergence may have rendered the SPARC-L1/−L2 proteins dispensable for enamel mineralization and other skeletal tissues. This strong redundancy might have allowed the occurrence of independent gene loss and of decreased expression levels that affected the *SPARC-L1/−L2* genes in distinct osteichthyan lineages.

*SPARC-L* is expressed during the enameloid late maturation stage in both examined chondrichthyans, which is similar to the timing of *Amtn*, *Odam* and *Scppq1* expression (all being SCPP genes) in various tetrapods ameloblasts [20, 65–69]. Hence, the ancestral gnathostome *SPARC-L* gene might first have been involved in the maturation of the outer odontode layer, without necessarily contributing to its protein composition per se. In this respect, the role of EMPs as the major architectural proteins of the enamel matrix, best exemplified by the more recent emergence of the *Amel* gene, may represent a sarcopterygian-specific evolutionary innovation [41]. Based on our results, we propose that all odontode external layers are homologous, and that secondary modifications (e.g. shifts in the expression timing of *EMP* genes in sarcopterygians, recruitment of the collagen/SPARC module in teleost and salamander ameloblasts) have led to a variety of derived structures known today as enamel, enameloid, ganoine or acrodin. In the future, describing *SPARC-L1* and *SPARC-L2* expression patterns in odontodes of non-tetrapod species, and unravelling the localization and function of chondrichthyan SPARC-L proteins during the mineralization process, will represent a major breakthrough in our understanding of vertebrate odontode evolution.

## Conclusions

We show here that the *SPARC* gene is conserved in all gnathostomes examined to date and that the chondrichthyan *SPARC-L* gene is co-orthologous to the osteichthyan *SPARC-L1* and *SPARC-L2* genes. We introduce the “*SPARC-L* gene family” nomenclature to describe the full repertoire of gnathostome *SPARC-L*, *SPARC-L1*, *SPARC-L2* and *SCPP* members, which are all derived from an ancestral gnathostome *SPARC-L* gene.

A highly conserved odontoblastic *collagen/SPARC* module confirms gnathostome dentin homology. The exclusion of the *collagen/SPARC* module from ameloblasts is a shared feature of the amphibian and the two elasmobranch species examined here, and of mammals. In addition, the ameloblastic expression of the single *SPARC-L* gene in chondrichthyans, similar to the *EMPs* in osteichthyans, probably best reflects the ancestral gnathostome situation. Taken together, our results provide new evidence in favor of a more general definition of the odontode external layer and support its homology in all gnathostomes.

## Methods

### Transcriptomic data

Sequences belonging to the *SPARC* and *SPARC-L1/−L2* gene families were obtained by screening jaw transcriptomes (Illumina sequencing, see [56] for taxon sampling details and reference to SRA IDs). These available transcriptomic data were assembled at ISEM-Montpellier (France) and subsequently screened using BLAST on the Montpellier Bioinformatics Biodiversity (MBB) platform (http://mbb.univ-montp2.fr/MBB//index.php). The species chosen in these transcriptomic data were: the catshark *Scyliorhinus canicula* (late embryonic stage 33, 9 cm total length (TL) and 20 cm TL juvenile); the thornback ray *Raja clavata* (late embryonic stage 33, courtesy of the Centre de découverte de la pêche en Mer (Maréis), Etaples sur Mer, France)*;* the salamander *Pleurodeles waltl* (6 month-old, reared in J-Y S. lab facility); two lungfishes: *Lepidosiren paradoxa* (juvenile, 10.0 cm TL, pet shop, Challet Herault) and *Protopterus aethiopicus* (juvenile, 12.0 cm TL, pet shop Challet Herault); the bichir *Polypterus senegalus* (2 year-old, reared in J-Y S. lab facility); the bowfin *Amia calva* (juvenile, 10.0 cm TL, pet shop, Challet Herault); the sturgeon *Acipenser baerii* (larvae, Sturgeon SCEA farm, France) and two gymnophionans *Typhlonectes compressicauda* and *Rhinatrema bivittatum* (juveniles, courtesy of Philippe Gaucher, CNRS French Guiana).

### Reconstruction of the SPARC/SPARC-L1/−L2 phylogeny

Sequences of the *SPARC* and *SPARC-L* gene families were obtained as first hits after similarity analyses (BLAST) using *SPARC* and *SPARC-L1* sequences of the mouse transcripts (NCBI references: BC004638.1 and NM_010097.4 respectively) against all available transcriptomes. New nucleotide sequences of the *SPARC* and *SPARC-L* gene families were deposited in Genbank under the IDs: MH206590 (*Lepidosiren paradoxa SPARC*), MH206591 (*Pleurodeles waltl SPARCL1*), MH206592 (*Pleurodeles waltl SPARC*), MH206593 (*Protopterus aethiopicus SPARC*), MH206594 (*Polypterus senegalus SPARCL2*), MH206595 (*Polypterus senegalus SPARC*), MH206596 (*Typhlonectes compressicauda SPARC*), MH206597 (*Rhinatrema bivittatum SPARC*), MH206598 (*Scyliorhinus canicula SPARCL*), MH206599 (*Raja clavata SPARC*), MH206600 (*Raja clavata SPARCL*), MH206601 (*Acipenser baerii SPARCL1*), and MH206602 (*Amia calva SPARCL1*). Phylogenetic analyses were performed to infer the evolutionary history of these two gene families and provide orthology assignments. Protein sequences coming from public databases and the jaw transcriptomic data (see Additional file 1) were aligned using MAFFT [70]. Members of the SCPP gene family were not included as they only share their 5′ sequence with *SPARC-L1*, which includes a small coding region. Regions of ambiguous sequence homology were removed with GBlocks [71] (−b1 21 -b2 21 -b3 16 -b4 5 -b5 half), generating a 235 amino-acid alignment in the complete set of sequences (Additional file 3) that has been analyzed without (Fig. 1 and Additional file 2: b) or with (Additional file 2: a, c) the amphioxus sequences. Both datasets were used to reconstruct gene phylogenies with either Bayesian or Maximum Likelihood approaches (Additional file 2). Bayesian inferences were run using Phylobayes 4,1 [72] with the site-heterogeneous CAT+Γ4 model of sequence evolution. Two independent chains were run for 10,000 cycles, and convergence was checked using pbcomp and tracecomp programs. A burnin of 50% was used to obtain the consensus tree (Fig. 1, Additional file 2: a). Maximum Likelihood analyses were run using IQ-TREE [73] with site-heterogeneous C20 + R4 + F model of sequence evolution. 100 bootstraps were performed and then mapped onto the best Maximum Likelihood tree (Additional file 2: b, c). Both models take into account the site-heterogeneity of substitution processes, which is particularly important given the peculiar characteristics of SPARC sequences (with highly conserved cysteine positions). Reconstructions were rooted with the amphioxus sequences when included (Additional file 2: a, c) or with the lamprey sequences (Fig. 1 and Additional file 2: b).

### Specimens, cryosections and histological sections

Lesser spotted catshark (*Scyliorhinus canicula*) embryos were obtained in Montpellier from the Station Méditerrannéenne de l’Environnement Littoral, Sète, France. Thornback ray (*Raja clavata*) embryos were kindly provided by the Centre de découverte de la pêche en Mer (Maréis), Etaples sur Mer, France. Embryos were raised in a smell tank at 18 °C until they reached the proper stages of tooth development (stage 33 as described in [74, 75]). Embryos were taken off the eggshell and euthanized following all European animal-care specifications, by overdose of MS-222 (Sigma), then fixed 48 h in PFA 4% in PBS 1× at 4 °C. They were finally transferred and stored in ethanol at − 20 °C.

Adult *Xenopus tropicalis* are maintained at the University of Concepcion, following standard protocols established for this species. Embryos and tadpoles were raised after natural mating and staged according to the Nieuwkoop and Faber developmental table [76]. Anesthesia of tadpoles was performed with a MS-222 (Sigma) solution at 200 mg/mL and specimen were subsequently decapitated in agreement with international bioethical recommendations [77, 78].

Catshark, thornback ray, and frog specimens were embedded in wax. 7 μm-thick histological sections were cut and then stained with standard protocols (eosin, hematoxylin and safran reaction for catshark and thornback ray sections (RHEM platform at IRCM, Montpellier); hematoxyline and chromotrope 2R (C3143 Sigma) for frog sections). Clawed frog in situ hybridizations were made on 7 μm thick paraffin sections of the upper jaw oriented longitudinally. Spotted catshark and thornback ray in situ hybridizations were performed on 14 μm thick cryostat sections (para-sagitally in lower jaws and transversally in the body trunk). Parts of the specimens that were not used for this study were conserved in ethanol at − 20 °C for further studies on gene expression.

### DIG-labeled riboprobe synthesis

Identified thornback ray *Col1a1*, *Col1a2*, *Col2a1*, *SPARC* and *SPARC-L1* sequences were used to design primers and amplify selected sequences from a cDNA extract obtained from the jaw of a stage 33 embryo (primer sequence given in Additional file 4). PCR products were ligated into the pGEM-Teasy vector using the TA cloning kit (Promega). Identified catshark *SPARC* and *SPARC-L* sequences were used to screen a cDNA library of catshark embryo RNA extracts [79] and one clone for each gene was isolated. Xt-Col1a1, Xt-Col1a2, Xt-Col2a1, and Xt-SPARC (GenBank NM_001011005.1; NM_001079250.1; NM_203889 and AY575077, respectively) were amplified from stage NF60 calvaria cDNA. Xt-Enam (GenBank NM_001145743) was amplified from stage NF60 upper jaws. All PCR products were blunt-cloned into the pBluescript vector (see Additional file 4 for primers). All inserts were amplified using primers present in the vector backbone or in the insert (see Additional file 4 for internal primers), and PCR products were used as a template to synthesize antisense DIG riboprobes in a 3 μl reaction containing 100–200 ng PCR product and using the DIG RNA labeling mix (Roche) and T7 or Sp6 RNA polymerase (Promega) following manufacturer’s instructions. DIG-labelled riboprobes were purified on MicroSpin G50 column (GE Healthcare).

### In situ hybridization and image processing

In situ hybridization of xenopus, catshark and thornback ray jaw sections were performed as described previously [59]. Images of the catshark in situ hybridization and histological staining were taken under a Hamamatsu NanoZoomer 2.0-HT Slide Scanner (Montpellier RIO Imaging facility, INM Optique) for the analysis of gene expression patterns. Slides were first scanned with a 20× objective and selected sections were scanned again with a 40× objective when necessary.

## Additional files

Additional file 1: *SPARC* and *SPARC-L* sequences used in the phylogenetic reconstruction. (XLSX 10 kb)

Additional file 2: Bayesian (a) and Maximum Likelihood (b, c) trees. Amphioxus sequences are included in a and c, excluded in b. (PDF 722 kb)

Additional file 3: Protein sequence alignment – 235 amino-acids. (PHY 15 kb)

Additional file 4: List of primers used in this study. (DOCX 17 kb)

## Abbreviations

EMP: Enamel Matrix Proteins
*R.c.*: *Raja clavata*
*S.c.*: *Scyliorhinus canicula*
SCPP: Secretory Calcium-binding PhosphoProtein
SIBLING: Small Integrin-Binding LIgand, N-linked Glycoproteins
SPARC: Secreted Protein Acidic and Rich in Cysteine
*X.t.*: *Xenopus tropicalis*
