# Supplementary figures and images for: Evolution of dental tissue mineralization: an analysis of the jawed vertebrate SPARC and SPARC-L families
Source: BMC Evol Biol. 2018 Aug 30;18:127. doi: 10.1186/s12862-018-1241-y (PMC6117938; doi:10.1186/s12862-018-1241-y)

Supplementary material 2a

0.2

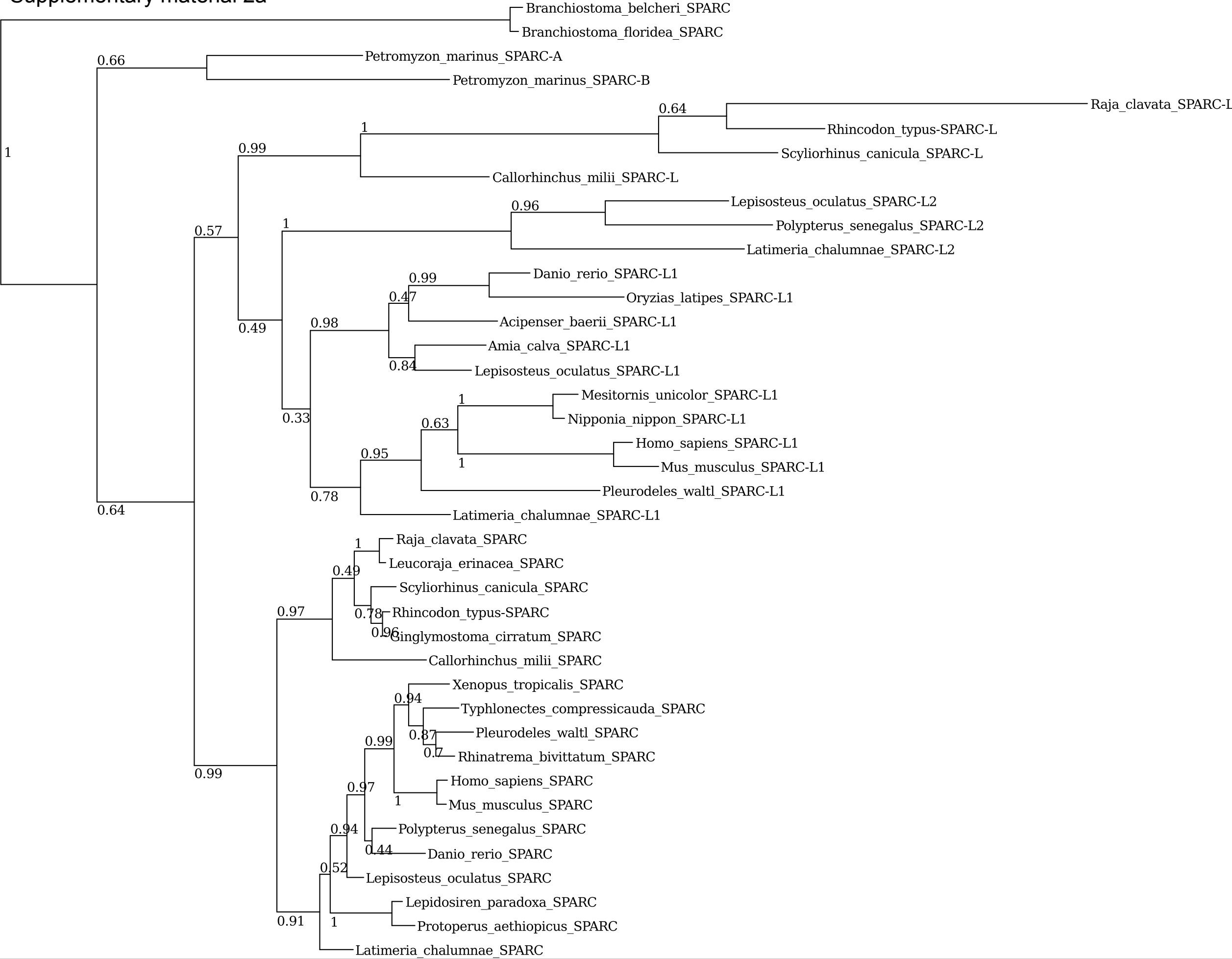

Supplementary material 2b

0.2

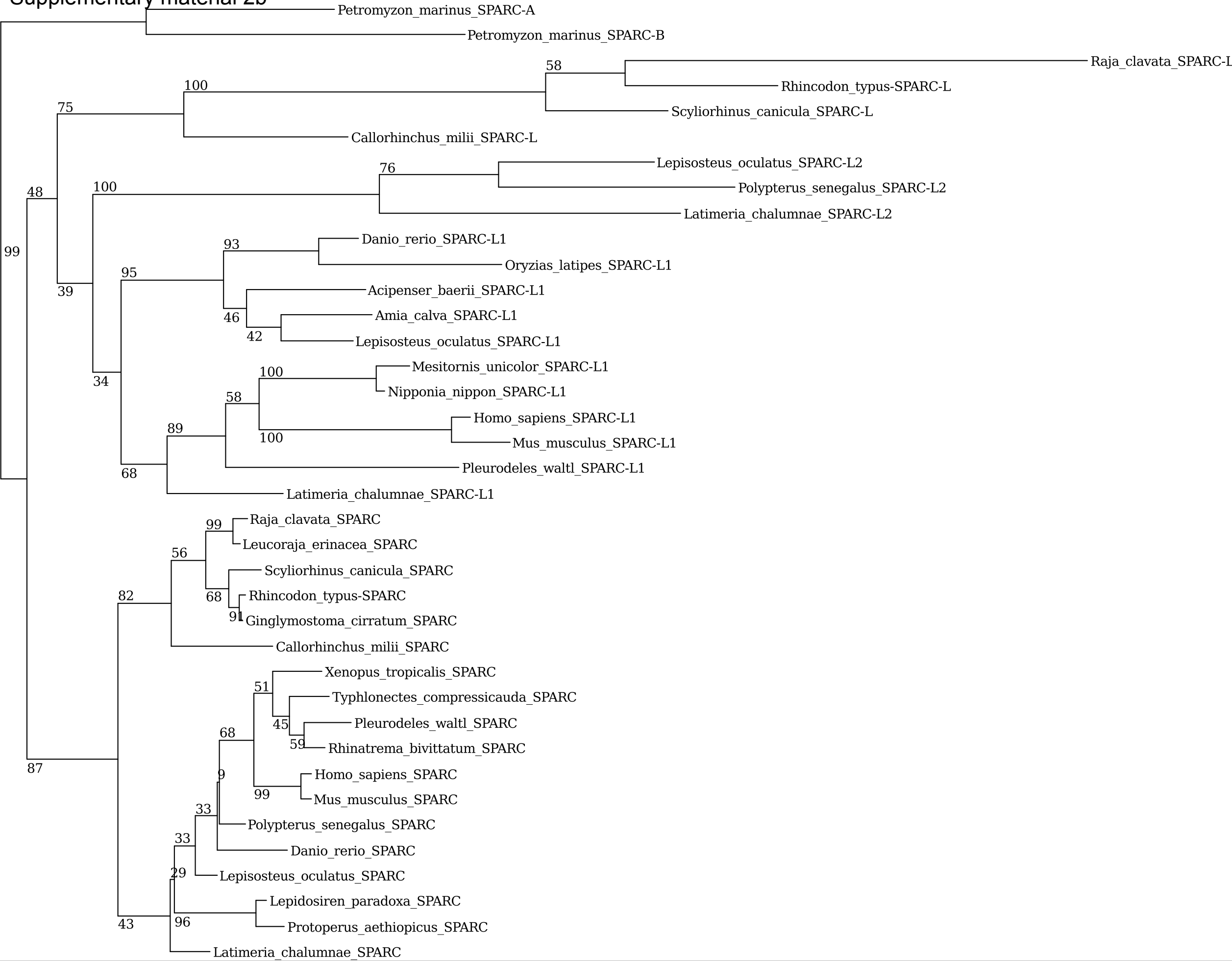

Supplementary material 2c

0.2

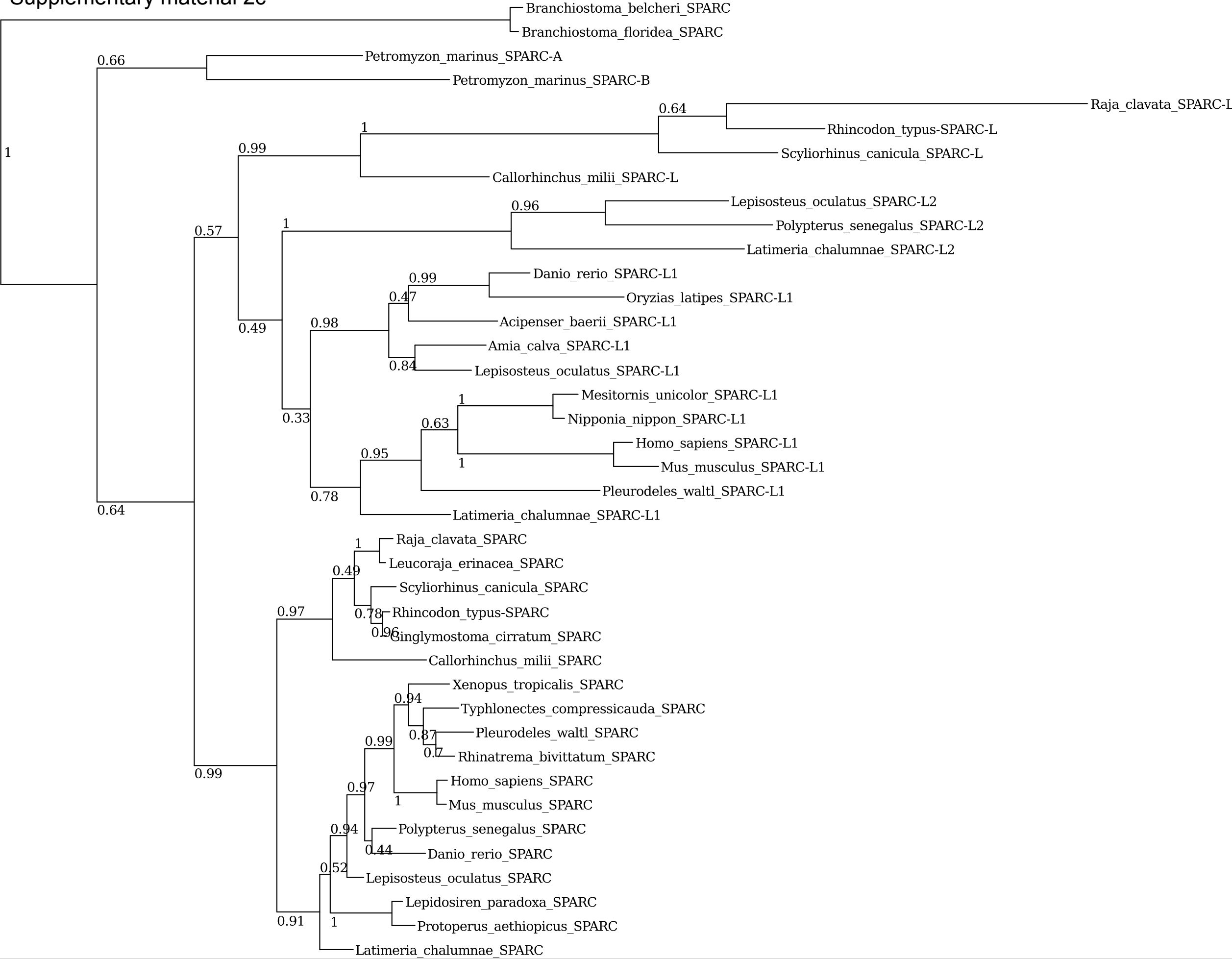

Supplement: Supplementary file 2 — Bayesian (a) and Maximum Likelihood (b, c) trees. Amphioxus sequences are included in a and c, excluded in b. (PDF 722 kb) [file 12862_2018_1241_MOESM2_ESM.pdf]
