# Supplementary material for: Evolution of dental tissue mineralization: an analysis of the jawed vertebrate SPARC and SPARC-L families
Source: BMC Evol Biol. 2018 Aug 30;18:127. doi: 10.1186/s12862-018-1241-y (PMC6117938; doi:10.1186/s12862-018-1241-y)
Supplement: Supplementary file 4 — List of primers used in this study. (DOCX 17 kb) [file 12862_2018_1241_MOESM4_ESM.docx]

| **Amplified sequence** | **Species** | **PCR product length** | **Primer orientation** | **Primer name** | **Primer sequence** |
| --- | --- | --- | --- | --- | --- |
| *Col1a1* | *Raja clavata* | 653 | Forward | Rc-Col1a1-For | TGAACGAGGCAGTCCTGGA |
|  |  |  | Reverse | Rc-Col1a1-Rev | TGCAGACCTGGAGCACCTT |
| *Col1a2* | *Raja clavata* |  | Forward | Rc-Col1a2-F | TTTGGTCCTCAAGGAAACCC |
|  |  |  | Reverse | Rc-Col1a2-R | CTGATTCACCTCTCGTTCCT |
| *Col2a1* | *Raja clavata* | 595 | Forward | Rc-Col2a1-For | ATGCCTGGTCCTCAAGGT |
|  |  |  | Reverse | Rc-Col2a1-Rev | CAGCCCTGGTTAGGATCG |
| *SPARC* | *Raja clavata* |  | Forward | Rc-SPARC-F | GTGGCACTGATAACAGCACA |
|  |  |  | Reverse | Rc-SPARC-R | GAAGGTTGCCATCAATATCC |
| *SPARC-L* | *Raja clavata* |  | Forward | Rc-SPARC-like-F | TCTGTGGCACCAACAACGA |
|  |  |  | Reverse | Rc-SPARC-like-R | GGGATCAATGTCCTCTTCT |
| *SPARC* | *S. canicula* |  | Forward | Sc-SPARC-E106-internFor | CAAAGGAGCACTGCACTACT |
|  |  |  | Reverse |  | n/a |
| *SPARC-L* | *S. canicula* | 1000 | Forward | Sc-SPARCL-F20-intF2 | TGAGCACCATCTTCACCCTC |
|  |  |  | Reverse |  | n/a |
| *Col1a1* | *Xenopus tropicalis* | 1197 | Forward | *XtCol1a1-F* | GGCACCCATGGATATCGGAG |
|  |  |  | Reverse | *XtCol1a1-R* | CGCTGTCAACTTTTGGGTCG |
| *Col1a2* | *Xenopus tropicalis* | 669 | Forward | *XtCol1a2-F* | GAGGGCAACAGCAGATTCAC |
|  |  |  | Reverse | *XtCol1a2-R* | ACGGAAAAGTGAGTCGTAAGC |
| *Col2a1* | *Xenopus tropicalis* | 901 | Forward | *XtCol2a1-F* | GGCTGCAAGAAACACACTGG |
|  |  |  | Reverse | *XtCol2a1-R* | CTCCCAGATGCAGAACCCAG |
| *SPARC* | *Xenopus tropicalis* | 1045 | Forward | *XtSPARC-F* | AGGTCCGACTTCAGAATGAG |
|  |  |  | Reverse | *XtSPARC-R* | TCTAAGTTAGCACCTTTGCTG |
| *Enam* | *Xenopus tropicalis* | 812 | Forward | *XtEnam-F* | CCCATGGCTCAGAATGGCTA |
|  |  |  | Reverse | *XtEnam-R* | TCAGGTAAATCAGGCTCCGG |
